# Reimagining Nursing Education and Reinvesting the Dividends to Build Healthier and More Equitable Communities

**DOI:** 10.1111/jan.70418

**Published:** 2025-12-07

**Authors:** Robert Atkins

**Affiliations:** ^1^ Johns Hopkins University School of Nursing Baltimore Maryland USA

One of the most pivotal developments in health and healthcare over the past 50 years was the 1974 publication of the Lalonde (1974) Report, “A New Perspective on the Health of Canadians.” This groundbreaking report initiated a paradigm shift by recognizing that health and well‐being are influenced not only by clinical care but also by the environments in which individuals live, work, play, and learn. Lalonde's report redefined the concept of health and sparked a worldwide movement to address social determinants, integrating them into research, policy, and practice. This evolution continues to impact health systems today, underscoring the necessity of interventions beyond clinical settings, including within homes, schools, workplaces, and communities.

Lalonde's report echoed the early vision of nursing pioneers such as Lillian Wald and Mary Breckenridge. Wald established the Henry Street Settlement in New York City, setting a precedent for public health nursing, while Breckenridge founded the Frontier Nursing Service in Kentucky, bringing accessible care to rural America. Their visionary work laid the foundation for integrating social determinants and community engagement into nursing practice. However, as hospitals proliferated in the early twentieth century, nursing education and practice shifted toward clinical care within hospital walls, which sidelined the community‐based roots of the profession (Atkins 2024).

The 1974 publication of the Lalonde Report, “A New Perspective on the Health of Canadians,” was a watershed moment that catalyzed a paradigm shift in health and healthcare, broadening the focus beyond clinical care to encompass the social determinants that shape well‐being. In a similar vein, Patricia Benner's Educating Nurses: A Call for Radical Transformation (2010) initiated a paradigm shift within nursing itself and profoundly transformed nurse education. By challenging traditional, time‐based educational models and championing competency‐based education (CBE), Benner's work reoriented the profession toward mastery of essential skills and knowledge, equipping nurses to meet the demands of increasingly complex health and healthcare environments.

To be certain, there are significant health and healthcare challenges that persist today, such as limited access to care, rising healthcare costs, workforce shortages, and disparities in health outcomes among different populations. These challenges are further complicated by shifting demographics, technological advancements, and the growing burden of chronic diseases. However, there is good reason to believe that our capacity to address these issues increases as we focus more intentionally on the upstream determinants of health and better leverage the unique skills that nurses bring as providers, problem‐solvers, and leaders.

In the following pages, I explore how reimagining nursing education can be shaped by the transformative ideas found in the Lalonde Report and Benner's call to action. These perspectives guided the recommended changes needed to address both current and future challenges. Specifically, nursing education must fully integrate competency‐based education—including robust clinical rotations—leverage its dividends to expand and diversify the workforce, and create an educational architecture that centers communities and the social determinants of health.

The next section of this paper explores how integrating competency‐based education with robust clinical rotations is a critical step in this larger redesign. By aligning educational structures with the realities and complexities of patient care we can ensure that our graduates are prepared, adaptive, and ready to lead in an ever‐changing healthcare landscape.

## 1 Fully Embrace Competency‐Based Education

The American Association of Colleges of Nursing (AACN) Essentials: Core Competencies for Professional Nursing Education, published in 2021, represents a foundational shift in the way nursing is taught and learned in the United States (AACN 2021). These Essentials were developed in direct response to persistent calls for reform—most notably those articulated by Benner and colleagues in their seminal work, which argued that nursing education required a radical transformation to overcome the limitations of traditional, time‐based curricula and to better prepare nurses for the realities of clinical practice.

Unlike older models that primarily measured progress by credit hours and time spent in classrooms or clinical placements, the AACN Essentials framework outlines explicitly defined domains and subdomains of practice, requiring students to demonstrate mastery of each competency before advancing (AACN 2021). This competency‐based approach is a marked disruption from convention, shifting the focus from exposure to content toward proven ability in practical, real‐world scenarios.

This transition toward competency‐based education (CBE) is especially promising as it directly tackles one of nursing education's most enduring critiques—the academic‐practice gap—a problem rigorously documented by Benner et al. (2010). CBE is designed to ensure that all competencies are measurable, observable, and aligned with the complex skills nurses need in practice, as noted by (Atkins and Szanton 2025; Atkins, Brown, Mudd, et al. 2025; Atkins, Brown, and Szanton 2025). By emphasising outcome‐driven learning, CBE ensures that graduates emerge not only as knowledgeable and clinically competent professionals but also as practitioners capable of translating learning into effective patient care.

Yet, while the shift to CBE represents a meaningful advance, it is important to recognise that the transformation cannot be considered comprehensive until clinical rotations are fully integrated into the competency framework. As we argued in our recent manuscript (Atkins, Brown, and Szanton 2025), the true value of CBE is realised only when students have regular, structured opportunities to apply their competencies in authentic clinical settings. Clinical rotations are essential for bridging the gap between classroom theory and real‐world practice—they provide the context in which students can demonstrate, refine, and internalise the competencies outlined in the AACN Essentials. Without these hands‐on experiences, CBE risks becoming another academic exercise, rather than a robust preparation for the complexities of nursing practice.

Competency‐Based Education offers a transformative approach to nursing clinical rotations by shifting the focus from time‐based experiences to the demonstration of clearly defined, practice‐ready competencies. The persistent reliance on traditional models—where students accumulate clinical hours without assurances of consistent or meaningful exposure—perpetuates variability in readiness for practice (Atkins, Brown, and Szanton, under review). CBE directly addresses this by providing students with explicit competencies that are observable, measurable, and closely aligned with professional nursing roles. For example, at the start of a clinical rotation, students might receive a digital dashboard outlining essential competencies such as safe medication administration, patient communication, and clinical documentation. These benchmarks guide both learning and assessment, ensuring that students and preceptors share a transparent understanding of expectations throughout the rotation.

In sum, a crucial step in advancing nursing education is to ensure that competency‐based curricula include well‐designed clinical rotations, allowing students to practice and be assessed in the environments where their skills will matter most. This approach not only strengthens the alignment between education and practice but also ensures that new nurses are truly prepared to meet the demands of today's dynamic healthcare landscape.

## 2 Use CBE Dividends to Expand and Diversity Nursing Workforce

As highlighted in Atkins, Brown, and Szanton (2025) clinical rotations represent an incredibly heavy operational and programmatic weight for nursing programs, requiring substantial faculty resources and coordination while also demanding a significant investment of students' time. On average, nursing students in the United States spend between 600 and 1000 h in clinical settings over the course of their education, depending on program requirements and state regulations (Spector et al. 2025). It is essential to recognize that clinical rotations, while invaluable for hands‐on learning, also carry an inherent opportunity cost. Every hour students dedicate to clinical placements is time not available for engaging in other forms of learning such as mastering didactic course work and simulation‐based education (SBE).

The comprehensive transition to competency‐based education (CBE) is more than an academic reform—it's a change in investment strategy in the future of nursing. By shifting the investment from accumulating clinical hours to targeted competency development, CBE enables students to acquire essential skills and knowledge more efficiently. This purposeful approach replaces chance encounters with meaningful clinical experiences, guiding students to master the competencies required for practice readiness.

Importantly, by investing in CBE to improve efficiency in both classrooms and clinical settings, nursing programs will realize significant returns—what might be called the “dividends of CBE.” These dividends will include streamlined learning pathways, reduced reliance on rigid time‐based models, and the capacity to admit and graduate more students without compromising educational quality. Not only will this benefit students, but it also has profound implications for creating an architecture of nursing education that relieves the persistent bottleneck afflicting nursing programs. Programs have long struggled to admit all qualified applicants due to shortages of clinical placements and faculty, leading to missed opportunities for aspiring nurses (AACN 2022). With CBE, clinical rotations become more strategic and resource‐efficient, allowing programs to expand access and admit a greater number of applicants.

Our work at the Johns Hopkins University School of Nursing illustrates how reinvesting CBE dividends can optimise clinical education and drive diversity. Through the Outside Track initiative, we are exploring how clinical rotations with RN preceptors outside traditional acute care settings can support students' practice readiness (Johns Hopkins School of Nursing 2024). These experiences expose students to a broader spectrum of healthcare contexts and patient populations, enhancing their adaptability and understanding of real‐world nursing practice. Concurrently, the Johns Hopkins Clinical Experience pilot investigates the impact of employing unit‐based registered nurses as preceptors—both in individualised and small‐group models—on student preparedness for entry into the nursing workforce. These innovative pilot programs not only expand the capacity for clinical education but also foster more personalised learning, enabling preceptors to identify and address competency gaps and mentor students from diverse backgrounds.

Rather than using these gains simply to maintain the status quo, the dividends from CBE should be strategically reinvested to create a new architecture for nursing education—one that not only increases the number of nursing graduates but also intentionally prioritizes diversity within the workforce. Research demonstrates that a diverse nursing workforce more effectively addresses the health needs of all populations (Ku and Vichare 2023). This reinvestment might take the form of expanding targeted recruitment to enroll students from historically underrepresented backgrounds, providing tailored mentorship and support during and after clinical rotations, and designing curricula that reflect the needs and realities of individuals and families living in communities nurses will serve.

With CBE, the roles of faculty shift from managing clinical groups to mentoring individual students, fostering deeper learning through personalized feedback and relationship‐building. At the same time, programs can leverage experienced RNs as preceptors, which not only enhances the educational experience but also expands the program's overall capacity. For instance, with approximately 4,000,000 RNs nationwide, nursing programs have a substantial pool of potential preceptors to recruit, train, and employ. By reimagining clinical education in this way—and by integrating initiatives like the Outside Track and Johns Hopkins Clinical Experience pilots—programs can produce graduates who are truly practice‐ready while simultaneously strengthening the pipeline for future nurses and addressing the national nursing shortage.

Additionally, as programs admit greater numbers of students and reduce the time and resources required for traditional clinical education, they are presented with a transformative opportunity: to actively expand and diversify the pipeline into the nursing profession. Competency‐based education, with its individualized approach, enables the identification and development of each learner's distinct strengths (Atkins, Brown, Mudd, et al. 2025). By prioritizing recruitment and tailored support for students from historically underrepresented backgrounds, programs contribute to a workforce that more accurately reflects the diversity of the communities it serves and is equipped to provide culturally competent care.

Ultimately, the returns generated by CBE are not just about producing more nurses—they are about transforming nursing education to be more inclusive, adaptive, and responsive to the needs of patients and communities nurses serve. By intentionally reinvesting these dividends, and continuing to innovate through initiatives like Outside Track and the Johns Hopkins Clinical Experience pilot, we lay the groundwork for a future in which nursing graduates are not only practice‐ready but also prepared to advance health equity, expand access to care, and meet the evolving needs of our society.

## 3 Centering Communities, Working Upstream, RN Role Expanded

As our understanding of what drives health and well‐being evolves, it becomes imperative for nursing education to adapt accordingly. This shift echoes the insights of the Lalonde Report, which emphasised that health outcomes are profoundly influenced by social determinants such as environment, lifestyle, and biology, not just clinical care. Building on the dividends of competency‐based education and the importance of workforce diversity, in the third and final section I argue that the next critical step is to design an educational architecture that centers communities and prepares nurses to excel across the full spectrum of care settings—acute care, primary care, health promotion, and health maintenance—and I provide some examples of the kinds of community‐based care nurses are delivering.

Although most Americans are born in hospitals and many spend their final hours there, as argued in the Lalonde report, the majority of factors shaping our health and well‐being throughout life are rooted in our communities. From the environments we live in to the social connections we make, what happens outside hospital walls plays a far greater role in determining our overall health. This perspective highlights the need to reimagine the architecture of nursing education—so that nurses are fully prepared to provide clinical care but also recognize and address the community‐based influences on health. As Pittman (2019) argued, “The new epidemiological reality is that most health outcomes are determined by social and environmental factors, not just clinical care.”

This shift in understanding calls for nursing to return to its foundational role in community health. At the turn of the twentieth century, public health nursing pioneers like Lillian Wald sought to build healthier communities by establishing an “organic relationship with the neighborhood” and engaging with the realities of people's lives (Atkins 2024). Wald and her contemporaries built the foundations for nursing in the community by focusing on the social determinants of health: housing, nutrition, sanitation, and access to education. Their work demonstrated that nurses, deeply embedded within communities, were uniquely able to address both medical and social needs, supporting whole‐person wellness. Revisiting these roots is essential in today's epidemiological landscape, as nurses are positioned to lead efforts that bridge clinical expertise with community engagement, ensuring care reaches people where they live and addressing the full spectrum of factors that shape health outcomes.

There is a growing recognition that nurses are uniquely positioned to bridge clinical care and social supports, making them indispensable leaders in advancing health equity at the community level. According to the National Academies of Sciences, Engineering, and Medicine (2021) “The future of nursing lies not in expanding hospital roles, but in reimagining care delivery in homes, schools, and neighborhoods.” As outlined in the *RN Role Reimagined*, primary care shortages can be mitigated by empowering registered nurses to take on expanded roles. This approach includes RN‐led visits, chronic care management, and the implementation of standardized procedures—strategies designed to enhance access to and continuity of care within community settings (California Health Care Foundation 2017).

I have argued that the future of health and healthcare must center on communities and place greater emphasis on the social determinants that shape well‐being. Nurses are uniquely positioned to deliver care that extends beyond hospital walls, addressing upstream factors such as environment, education, and social support. However, our current approach to nursing education does not adequately prepare nurses to work in this future. Prelicensure programs remain largely focused on acute care and traditional clinical settings, with limited opportunities for students to engage with community‐based practice or develop the skills needed to address social determinants of health. Moreover, as discussed, nurses can provide a broader array of clinical care than is currently realized—including leading new‐patient visits, managing chronic conditions, conducting independent assessments, and coordinating care for complex cases (California Health Care Foundation 2017). With targeted training and expanded clinical authority, registered nurses can take on specialized functions such as wound care, diabetes education, and care management, as well as play a central role in team‐based models that improve access and efficiency.

To prepare nurses for the ever‐evolving demands of healthcare, we must rethink what and how we teach in both classroom and clinical environments. This means integrating curricula that emphasise community engagement, interprofessional collaboration, and advocacy, alongside robust clinical experiences in diverse settings—including homes, schools, and neighbourhoods. As I have argued, CBE offers a promising pathway to this future by shifting the focus from time‐based training to mastery of essential skills and knowledge. Through CBE, we can ensure that every nursing student demonstrates proficiency in areas such as population health, care coordination, and leadership, while also providing flexible, individualised learning pathways that reflect the realities of modern practice. By leveraging CBE, nursing education can produce graduates who are not only practice‐ready but also equipped to lead transformative change in communities, deliver expanded clinical care, and advance health equity for all.

## 4 Nurse Centered Models of Care

Across the United States and internationally, innovative nursing models are fundamentally reimagining how care is delivered in homes, schools, and neighborhoods. These approaches share a commitment to person‐centered, community‐based care that extends beyond clinical treatment to address prevention, social determinants of health, and empowerment. Preparing nurses for these roles requires a readiness for community‐oriented practice—an imperative in nursing education.

One example in the United States is the work of Sarah Szanton and colleagues at the Johns Hopkins School of Nursing. Their Neighbourhood Nursing initiative aims to provide universal, place‐based, and inclusive health and social care directly to homes and community anchors such as schools and libraries. Teams of nurses and community health workers deliver comprehensive care to people of all ages, focusing on prevention, health equity, and deep community engagement. Early results from pilot programs in Baltimore demonstrate reduced hospitalizations, improved health metrics, and strengthened community trust.

Costa Rica's community‐oriented primary health care system is a global exemplar of Neighbourhood nursing and the reimagined nursing role. The backbone of this system is the EBAIS (Equipo Básico de Atención Integral de Salud), multidisciplinary teams that provide comprehensive care to defined geographic areas (Rosero‐Bixby and Dow 2021). These teams conduct annual home visits, prioritize prevention, and integrate public health with primary care, achieving universal access and strong community engagement.

Another international example of engaging nurses in community‐focused care is the Buurtzorg model in the Netherlands which has garnered widespread attention for its innovative use of self‐governing nurse teams. Buurtzorg nurses deliver the full range of medical and supportive services to clients in their homes, emphasizing patient independence, continuity of care, and professional autonomy. Each self‐managed team is responsible for all aspects of care for 50–60 patients within a neighbourhood, working closely with families, primary care providers, and community resources. Buurtzorg's approach has resulted in high patient and employee satisfaction, lower costs, and better health outcomes compared to traditional models (Gray et al. 2015). Its success has led to adaptations in countries such as Japan, Norway, Sweden, the United Kingdom, and the United States.

Collectively, these models demonstrate that the future of nursing practice lies in the ability to deliver care that is deeply embedded in the community. They highlight the need for educational reforms that prepare nurses for interprofessional collaboration, community engagement, and adaptability to diverse settings—ensuring readiness for the evolving demands of community‐oriented practice.

## 5 Conclusion

In this paper I have argued for a fundamental redesign of the architecture of nursing education—namely a comprehensive integration of competency‐based education. In addition, while I have focused on CBE in this paper, Artificial Intelligence (AI) and Simulation‐based Education (SBE) are important pillars of the redesigned architecture of nursing education (see Atkins and Szanton 2025; Atkins, Brown, Mudd, et al. 2025). It is important to acknowledge that the adoption of CBE, AI, and SBE within both classroom and clinical settings will necessarily be incremental, requiring thoughtful, phased implementation. Nonetheless, I believe that these investments will yield significant dividends for the nursing profession and the communities we serve.

I have aimed to provide a clear discussion of how I believe these resources and efforts should be strategically invested—expanding and diversifying the nursing workforce and centering education on preparing nurses to address social determinants of health and deliver acute and primary care in communities. Building on the foundational insights of the Lalonde Report, which emphasized the critical importance of factors beyond traditional healthcare—such as environment, lifestyle, and social supports—in shaping health outcomes, and Benner's call for a radical transformation in nursing education to better prepare nurses for these expansive roles, there is a growing recognition that nurses are uniquely positioned to bridge clinical care and social supports, making them indispensable leaders in advancing health equity at the community level.

This redesign lays the foundation for a healthcare system that is more equitable, accessible, and responsive to the needs of all communities. These reforms optimise nurses' strengths to address the social determinants of health, reduce disparities, and promote resilience at the community level. By fundamentally redesigning nursing education, we are strategically positioning the profession to leverage its considerable strengths—not only as the largest share of the healthcare workforce, but also as the most trusted professionals with a longstanding legacy of serving in communities. Nurses have consistently played a pivotal role in addressing the social determinants of health and delivering both acute and primary clinical care directly where people live, learn, and work. This approach ensures that the redesign maximises nursing's potential to drive positive health outcomes and advance equity at the community level. A diverse, practice‐ready nursing workforce is uniquely positioned to advance health equity, expand access to care, and foster trust between healthcare providers and the populations they serve. Ultimately, the transformation of nursing education is inseparable from the broader goal of improving community health and social well‐being.

## Funding

The author has nothing to report.

## Disclosure

All figures, tables, and materials reproduced from other sources have been used with permission and are properly cited.

## Ethics Statement

The author has nothing to report.

## Conflicts of Interest

The author declares no conflicts of interest.

## Data Availability

Data sharing not applicable to this article as no datasets were generated or analysed during the current study.
